# Myo-Inositol as a Key Supporter of Fertility and Physiological Gestation

**DOI:** 10.3390/ph14060504

**Published:** 2021-05-25

**Authors:** Riccardo Gambioli, Gianpiero Forte, Giovanni Buzzaccarini, Vittorio Unfer, Antonio Simone Laganà

**Affiliations:** 1 R&D Department, Lo.Li. Pharma, 00156 Rome, Italy; r.gambioli@lolipharma.it (R.G.); g.forte@lolipharma.it (G.F.); 2 Unit of Gynecology and Obstetrics, Department of Women and Children’s Health, University of Padua, 35128 Padua, Italy; giovanni.buzzaccarini@gmail.com; 3 The Experts Group on Inositol in Basic and Clinical Research (EGOI), 00161 Rome, Italy; antoniosimone.lagana@uninsubria.it; 4 System Biology Group Lab, 00161 Rome, Italy; 5 Department of Obstetrics and Gynecology, “Filippo Del Ponte” Hospital, University of Insubria, 21100 Varese, Italy

**Keywords:** inositol, myo-inositol, pregnancy, fertility, PCOS, ART, GDM, NTDs

## Abstract

Pregnancy is a complex process, featuring several necessary changes in women’s physiology. Most women undergo healthy pregnancies; even so, several women experience reduced fertility or pathologies related to the pregnancy. In the last years, researchers investigated several molecules as promoters of fertility. Among all, myo-inositol (myo-ins) represents a safe compound that proved useful in issues related to fertility and pregnancy. In fact, myo-ins participates in several signaling processes, including the pathways of insulin and gonadotropins, and, therefore, it is likely to positively affect fertility. In particular, several clinical trials demonstrate that its administration can have therapeutic effects in infertile women, and that it can also be useful as a preventive treatment during pregnancy. Particularly, myo-ins could prevent the onset of neural tube defects and the occurrence of gestational diabetes mellitus, promoting a trouble-free gestation. Due to the safety and efficiency of myo-ins, such a treatment may also substitute several pharmaceuticals, which are contraindicated in pregnancy.

## 1. Introduction

### 1.1. Background

Pregnancy represents a delicate period during the life of a woman. From its pursuit to the delivery, several criticalities may emerge, even if the greater part are nowadays preventable or manageable by applying a correct treatment plan. Inositols are important molecules in promoting female fertility and in sustaining physiological pregnancy. They are a family of six-membered carbocyclic polyalcohols, with nine possible stereoisomers, among which myo-inositol (myo-ins) and d-chiro-inositol (d-chiro-ins) have an established role in human physiology. Myo-ins represents the most abundant inositol isomer in mammals, and it is commonly found in almost every tissue, particularly in brain, blood, fat, kidney, lung, ovaries, and testes, where it participates in several cellular pathways. d-chiro-ins is the second most represented isomer, as it is commonly detectable as a minor constituent in almost all the tissues containing myo-ins [1]. Researchers found a higher myo-ins/d-chiro-ins ratio in tissues requiring high energy, and lower ratios in tissues where glucose is mainly stored as glycogen [2,3]. In the form of inositol phosphates, both isomers are primarily involved in cellular signaling cascades [3], transmitting extracellular stimuli to cellular organelles [4].

Inositol cascade is primarily involved in the signaling of insulin [5], gonadotropins [6,7], and in phenomena of cytoskeletal rearrangement [8]. Thus, in physiology, inositols represent the mediators of important functions, including cellular energetic metabolism, follicle maturation with menstrual cycle progression, and cellular motility. In particular, follicle maturation is a crucial process in fertility [9]; energetic dysmetabolism and correct glucose levels in blood are necessary for maintaining a physiological pregnancy [10]; and correct cellular motility is indispensable during processes of embryogenesis, such as neural tube closure [11].

### 1.2. Inositol in Insulin Signaling

**Figure 1:**
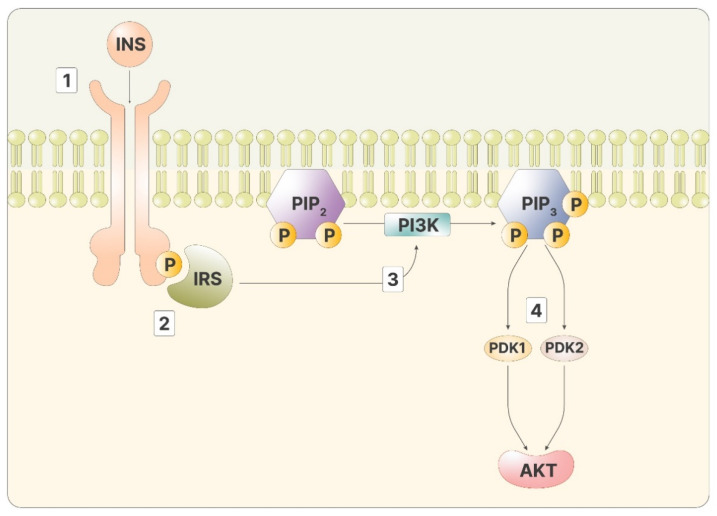
The figure depicts the intracellular cascade stimulated by insulin: 1—Insulin binds to its ligand; 2—IRS recognizes the phosphorylated receptor; 3—Activated IRS promotes the activity of PI3K, producing PIP3; 4—PIP3 stimulates both PDK1 and 2, activating Akt.

As mentioned, inositol phosphates are required to transmit the signal of insulin in the cytoplasm. Insulin signaling initiates when the hormone binds to the insulin receptor, a transmembrane tyrosine kinase receptor. Through kinase activity, the two intracellular domains of the receptor phosphorylate each other, improving the affinity for other ligands. The insulin receptor then phosphorylates the insulin receptor substrate (IRS), two proteins bound to the inner part of the membranes. Phosphorylated IRS is a ligand for PI3K, which, once bound, can catalyze the reaction that produces phosphatidyl-inositol-3,4,5-trisphosphate (PIP3) from the membrane lipid phosphatidyl-inositol-4,5-bisphosphate (PIP2) [12]. PIP2 can be cut by phospholipase C (PLC), releasing inositol-1,4,5-trisphosphate (IP3). High IP3 levels lead to a phosphorylation cascade of cytoplasmatic proteins, transporting the message from the membrane to the inner part of the cell. On the membranes, PIP3 activates Akt, via phosphoinositide-dependent kinase 1 and 2 (PDK1 and PDK2) (Figure 1).

**Figure 2:**
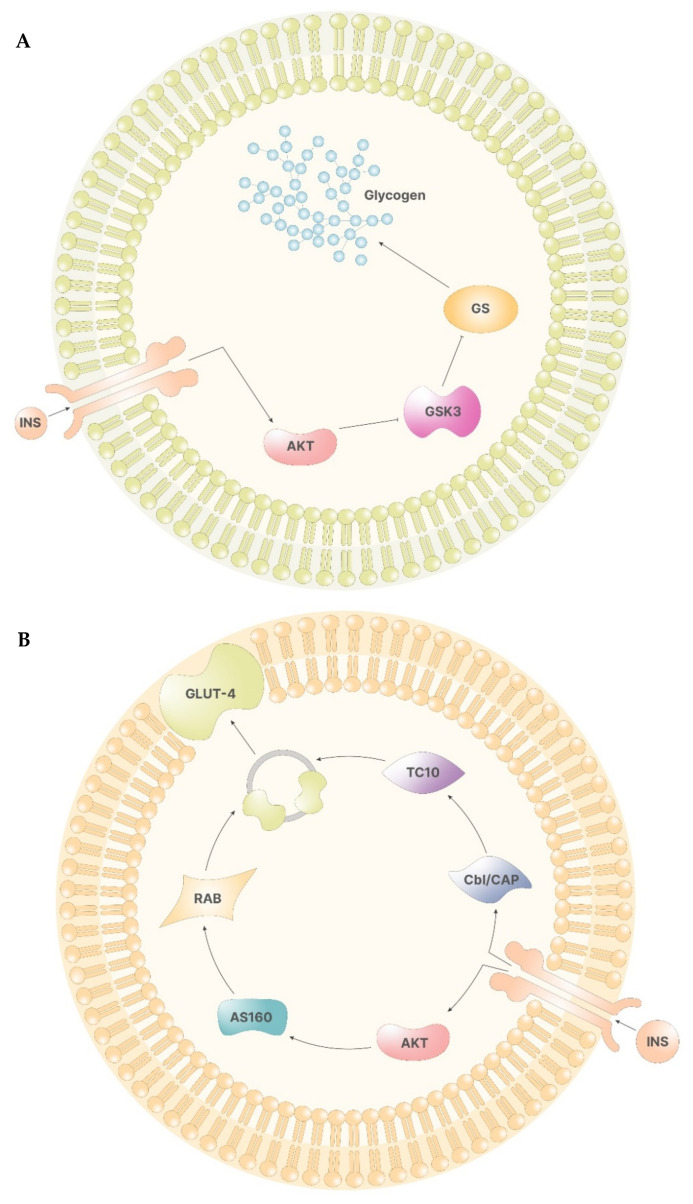
(**A**)—The signal of insulin leads to the activation of Akt via PIP3. In the liver cells, Akt inhibits the function of GSK3, which, if functional, would inhibit GS. Therefore, Akt activation leads to glycogen synthesis. (**B**)—In non-storage tissues, the activation of Akt enables the activity of AS160. AS160 activates Rab, which, in turn, promotes the formation of GLUT-4 containing vesicles. On the other side, the insulin receptor activates Cbl/CAP complex, which, via TC10, promotes the release of the vesicles containing GLUT-4.

In the liver, Akt, in turn, phosphorylates glycogen synthase kinase 3 (GSK-3), preventing the inhibition of glycogen synthase (GS). Hence, following insulin stimulus, inositol cascade induces the synthesis of glycogen in the liver (Figure 2A) [12].

In other tissues, the activation of the insulin receptor promotes the release of the vesicles containing glucose transporters (GLUT-4), which are responsible for cellular glucose uptake. Here, Akt mediates the direct release of vesicles by activating several proteins, including two atypical protein kinase C (PKC-ζ and PKC-γ) and AS160, which, in turn, activates Rab, a family of proteins involved in vesicle formations. On the other hand, phosphorylation of Cbl/CAP complex by the insulin receptor indirectly mediates vesicle release by activating TC10, a Rho family protein that induces GLUT-4 vesicles translation (Figure 2B) [12].

### 1.3. Inositol in Gonadotropin Signaling

**Figure 3:**
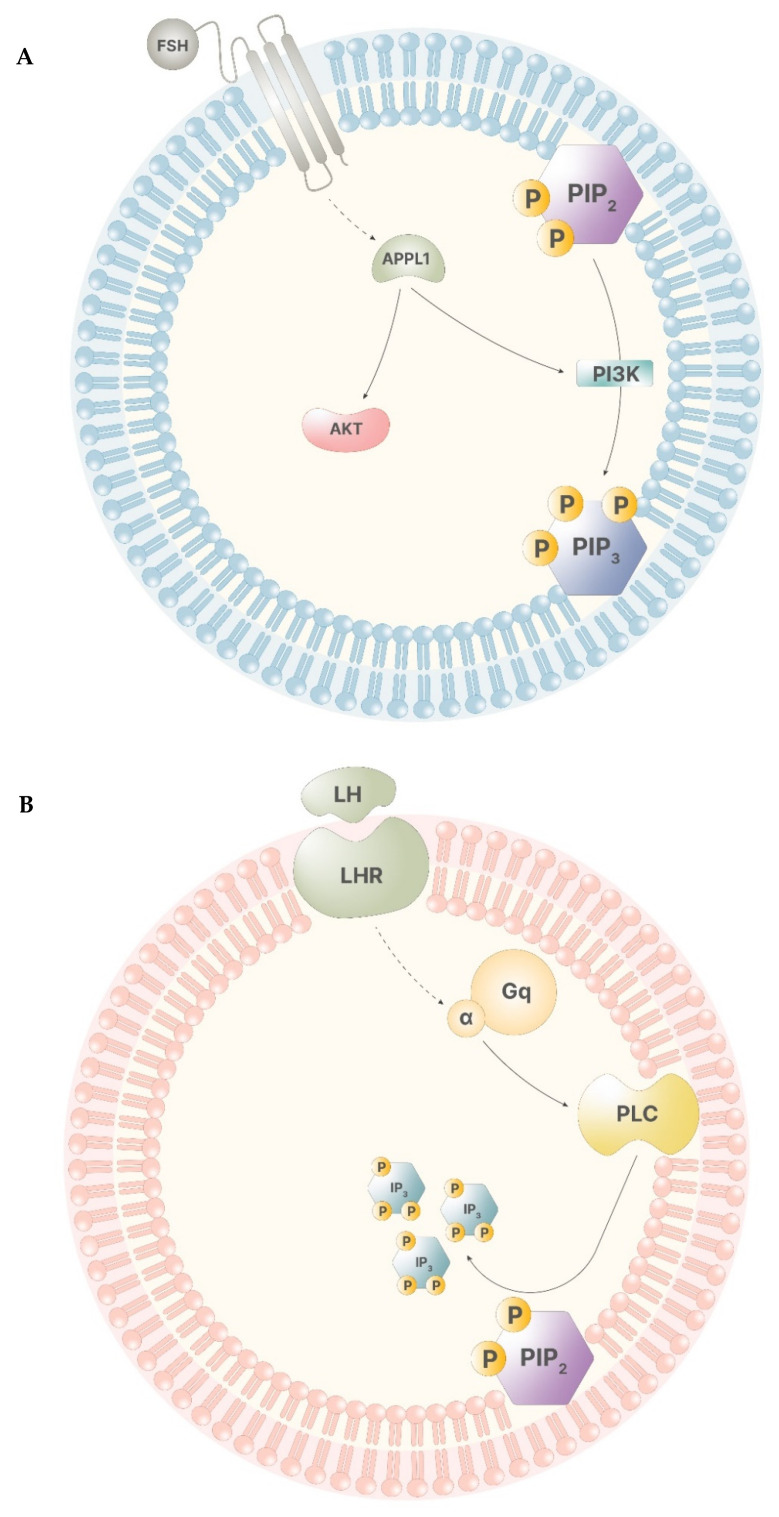
(**A**)—The receptor of FSH has low affinity for APPL1, therefore, only high levels of FSH trigger APPL1 activation. APPL1 activates Akt and stimulates PI3K, leading to the production of PIP3. (**B**)—The LH receptor poorly stimulates the activity of the alpha subunit of the Gq proteins. Thus, high LH levels lead to the activation of PLC that catalyze the cleavage of PIP2, producing IP3.

During a physiological menstrual cycle, signals from the hypothalamus–pituitary–gonadal (HPG) axis promote the progression of folliculogenesis through the stimuli of follicle stimulating hormone (FSH) and luteinizing hormone (LH). The synergistic activity of these two gonadotropins regulates gonadal functions, both in men and in women. Particularly, in women, FSH stimulus leads to the start of the menstrual cycle, allowing the cycle to enter into the follicular phase. FSH levels remains low until ovulation, when they rise, showing the highest peak that drastically falls with the end of ovulation. Until menses, FSH displays the lowest levels [6]. LH pattern of signaling is similar to that displayed by FSH. During the follicular phase, its levels appear to be low, rising drastically in preovulation, and falling to the basal level right after ovulation has occurred [7]. Myo-ins participates in both of these signaling pathways. When FSH binds to its receptor in the granulosa cells, the intracellular portion of the receptor develops a higher affinity for G proteins and APPL1, a leucine-zipper-containing protein that activates phosphatidyl-inositol-3-kinase (PI3K) and Akt, leading to transcriptional regulation and PIP3 production (Figure 3A). The inositol phosphate production is triggered only by elevated FSH levels, indicating the importance of inositol immediately before and during ovulation [6]. In thecal cells, LH receptor works in a similar way, activating a secondary pathway involving Gq protein alpha subunit, which, in turn, activates PLC, leading to IP3 production (Figure 3B). Of note, this process is triggered only by high levels of LH, indicating that this secondary pathway operates only before and during ovulation [7].

### 1.4. Inositol in Cellular Motility Phenomena

In neural tube diversification, both notochord and non-neural ectoderm are required for the correct progression of the process. Notochord ventral signaling allows medial hinge point formation and, thus, enables the beginning of the process. The activity of non-neural ectoderm is required during the closure phase. Non-neural ectoderm expresses Rac1, a small G protein involved in several cellular mechanisms, including cytoskeleton rearrangement, invasiveness, motility, and proliferation. Particularly, Rac1 promotes the convergent protrusion of non-neural ectoderm, which is required for neural tube closure, and its absence leads to spina bifida [11]. Rac1 activity is promoted by several proteins, including the kinase PKC-ζ [13] and Tiam1 [14], a protein bound to the plasma membrane responsible for GDP/GTP exchange activity. Tiam1 includes a recognizing domain for inositol phosphates, which can bind alternatively PIP2 or PIP3. Of note, PIP2 promotes Tiam1 tethering to the membranes, while PIP3 allows Tiam1 exchange activity, resulting in correct activity of Rac1 [14]. Hence, the binding of PIP3 to Tiam1 on the inner membranes activates Rac1, leading to the protrusions necessary during neural tube closure.

### 1.5. Opportunity for Inositol Supplementation

Inositol unbalance may lead to reduced fertility or pregnancy complications; thus, its administration can be useful in the prevention and in the treatment of several pregnancy-related pathologies. To restore fertility, current pharmaceutical treatments include selective estrogen receptor modulators (SERMs) and aromatase inhibitors, but they often lead to burdensome side effects, such as abdominal pain, fatigue, hot flashes, or nausea [15,16]. During pregnancy, major complications may emerge, threatening the health of the mother and the fetus. These conditions include gestational diabetes mellitus (GDM) and neural tube defects (NTDs), which arise from insulin resistance and altered cellular motility phenomena, respectively. In the case of pregnant women with insulin resistance, physicians usually suggest treatments with insulin sensitizers like metformin, even if contraindications exist [17]. To prevent incorrect neural tube closure, folate administration is nowadays the gold standard in clinical practice. However, some NTDs are not responsive to folate intake and are defined folate resistant, resulting in a minor portion of pregnant women still being at risk for NTDs despite folate administration. In such cases, a different approach to prevent NTDs is necessary [18].

Given inositol activities, the therapeutical and preventive effects of inositol supplementation have been widely investigated in different clinical pictures, including infertility and pregnancies at risk. This review of the literature aims to highlight the possible applications, the efficiency, and the safety of inositol administration before and during pregnancy. This report includes the most advanced evidence to date in animal models and clinical trials.

## 2. First Preclinical Evidence

Researchers investigated inositol efficiency in murine models. While most of the findings were further evaluated in humans, there is some interesting evidence without verification in clinical trials. Among them, the positive effects on mice with metabolic syndrome is particularly appealing. This model of metabolic syndrome is female mice heterozygous for a disruption in the endothelial nitric oxide synthase gene (eNOS+/−). Such a model is obtained by breeding eNOS−/− females with wild type males and feeding the heterozygous cubs with a four-week long, high fat diet, thus inducing the syndrome. These mice are characterized by increased body weight and blood pressure, other than high levels of insulin and glucose in the blood.

### 2.1. Metabolic Maternal Outcomes

Ferrari et al. [19] demonstrated that treating obese pregnant mice with myo-ins plus d-chiro-ins in 40:1 ratio, corresponding to the average physiological ratio detectable in the human serum, reduced weight gain. In parallel, treated eNOS+/− dams showed lower glycaemia and improved cardiovascular parameters. They bred eNOS+/− with metabolic syndrome or obese wild type females with wild type males. The dams received water (placebo) or myo-ins/d-chiro-ins dissolved in the water until sacrifice, at term. They found a significantly lower systolic blood pressure in eNOS+/− mice supplemented with inositol in relation to the placebo-treated dams (myo-ins/d-chiro-ins 138.52 ± 6.48 mmHg versus placebo 157.03 ± 7.79 mmHg), but they did not detect a similar result in wild type obese mice. Furthermore, they found a significant improvement in the area under the curve of the glucose tolerance test carried out in eNOS+/− dams (myo-ins/d-chiro-ins 17,512.5 ± 3984.4 versus placebo 29,687.14 ± 8258.7), without matching results in wild type obese mice. The authors observed a minor weight gain in the wild type obese mice treated with the combination of inositols in relation to the placebo-treated mice (myo-ins/d-chiro-ins 10.9 ± 0.5 g versus placebo 12.6 ± 0.6 g). Additionally, eNOS+/− dams treated with myo-ins/d-chiro-ins displayed lower leptin levels (16,985 ± 976.4 pg/dL) when compared to placebo-treated (24,181.9 ± 3128.2 pg/dL). Particularly, leptin is a marker of inflammation, strongly related to adipose tissue, that represents a risk factor for miscarriage in humans also. Treatment with both inositol isomers proved to reduce obesity-related inflammation, helping to prevent miscarriages and events of stillbirth.

### 2.2. Fetal Outcomes

Longo et al. [20] demonstrated that administration of myo-ins plus d-chiro-ins in 40:1 ratio improves fetal parameters, including weight gain, insulin condition, and cardiovascular parameters. They bred eNOS+/− females with wild type males, then treated the dams with myo-ins plus d-chiro-ins in a 40:1 ratio, or with water as placebo, dividing the offspring into four groups: wild type placebo (WT), eNOS+/− placebo (eNOS+/−), wild type inositol treated (WT INO), and eNOS+/− inositol treated (eNOS+/− INO). The authors sacrificed the puppies at ten weeks of age. Firstly, eNOS+/− INO mice weighed less in respect to eNOS+/−, 18.2 ± 0.5 g versus 22.6 ± 0.8 g, respectively. Secondly, the glucose tolerance test showed lower glucose levels at 60, 90, and 120 min, both in WT INO and in eNOS+/− INO mice, when compared to the mice with the same genotype born from placebo-treated mothers. Thirdly, WT mice displayed significantly lower systolic blood pressure compared to eNOS+/−, 142.34 ± 8.79 mm Hg and 169.05 ± 7.5 mm Hg, respectively. Inositol treatment, in this case, affected only WT mice, lowering the systolic blood pressure from 142.34 ± 8.79 mm Hg in WT to 110.15 ± 10.8 mm Hg in WT INO.

The authors also evaluated the muscular response to contractile and vasorelaxant treatments, phenylephrine and acetylcholine, respectively. In the eNOS+/− breed, eNOS+/− INO displayed decreased vascular contractile responses to phenylephrine in relation to mice born from untreated mothers. Following a treatment with an inhibitor of eNOS, WT INO displayed a reduction in contractile response to phenylephrine when compared to WT. This suggested that inositol administration may improve cardiovascular contractions independently from the genotype. On the other hand, the treatment with acetylcholine showed an altered response in WT, improved in WT INO. Likewise, the vasorelaxation was abolished in eNOS+/− mice, being improved in eNOS+/− INO. These data demonstrate that maternal treatment with inositols during pregnancy affects the fetus also after birth and in adulthood, improving metabolic and cardiovascular parameters.

## 3. Clinical Applications

### 3.1. Myo-Inositol in the Pursuit of Pregnancy

Myo-ins plays a key role in fertility, as it participates in the signaling cascade of the HPG axis. In fact, myo-ins is the second messenger of the follicle stimulating hormone (FSH) and luteinizing hormone (LH), involving a long-way transduction cascade from the pituitary gland to other body districts [6,7]. In particular, the correct signaling of FSH allows the maturation of follicles and regulates the selection of the dominant follicle [9]. Such processes also depend on insulin signaling, which is mediated by myo-ins. Indeed, myo-ins supplementation can reduce systemic levels of insulin in cases of hyperinsulinemia, which inhibits the progression of the menstrual cycle and reduces fertility. Insulin resistance and compensatory hyperinsulinemia often occur in women affected by polycystic ovary syndrome (PCOS), who display altered gonadotropin signaling [9] and, hence, infertility [21]. Because of these activities in the gonadotropin and the insulin pathways, several authors investigated the efficiency of myo-ins administration in achieving pregnancy in infertile PCOS and non-PCOS women, either for natural conception or during assisted reproductive technology (ART) protocols.

Results from clinical trials reported in Table 1 suggest the potential of myo-ins in inducing ovulation and in raising pregnancy rate. The randomized and controlled study by Gerli et al. [22] provided the first clinical evidence on the use of myo-ins in PCOS women to induce spontaneous ovulation. They investigated the efficiency of myo-ins (100 mg, twice per day) against placebo, finding that women treated with myo-ins were less likely to withdraw from the study. Moreover, the ovulation rate was 23% in patients from the myo-ins group and only 13% in the placebo group. However, the authors prescribed myo-ins in small quantities and as a component of a multivitamin, and, thus, further evidence was required. In a following study, Costantino et al. [23] treated PCOS women with a much higher dose of myo-ins (4000 mg/die), plus folic acid (400 mcg/die). They found significant differences in ovulation that occurred in 69.5% of the women treated with myo-ins plus folic acid, and in 21% of those treated only with folic acid. In the same study, the authors compared progesterone levels between the two groups, showing a significantly higher concentration peak in women treated with myo-ins (15.1 ng/mL versus 6.6 ng/mL). Their results highlighted the beneficial effects of higher myo-ins quantities in the short term, even if with a small sample size.

Papaleo et al. [24] analyzed restoration of the menstrual cycle and pregnancy achievement as the outcome. They treated PCOS women with amenorrhea with 2 g myo-ins, plus 200 mcg folic acid, twice per day for six months, and evaluated the trend in menstrual cycle restoration. Within their study sample, 88% of patients recovered at least one menstrual cycle, and 72% of them maintained regular ovulatory activity during the follow-up. During the study period, 40% of the patients achieved pregnancy, showing promising applications of myo-ins in fertility restoration. Even if the data appear promising, the small sample size and the absence of a control group reduce the potency of the study. Later, Raffone et al. [25] investigated the clinical pregnancy rate in PCOS women after ovulation induction with 4000 mg/die myo-ins, plus 400 mcg/die folic acid, compared to treatment with 1500 mg/die metformin. After the treatment, the authors treated nonpregnant ovulatory women from both groups with FSH (37.5 U/die). The authors found that myo-ins and metformin yielded similar results in pregnancy rate, 28.9% and 26.1% respectively, while, following stimulation, the total pregnancy rate raised to 48.4% among women treated with myo-ins and 36.6% among those treated with metformin. These results from a high-quality study suggest the strong usefulness of myo-ins in restoring fertility. More recently, Allah et al. [26] administered 2000 mg/die myo-ins to PCOS sub-fertile women for six months, checking their clinical features every trimester. After three months, 24.3% of patients displayed a regular menstrual cycle, while 38.6% of the women achieved ovulation. At the end of the study, 53.6% of patients achieved a regular menstrual cycle, with an increase to 72.1% of the patients who ovulated. As for the study by Papaleo et al., the absence of a control group affects the quality of the data. A meta-analysis by Pundir et al. [27] underlined the efficiency of myo-ins in restoring fertility in PCOS women, highlighting that previous data show a trend towards ovulation induction with myo-ins. In addition, their analysis highlights that myo-ins proved to restore the physiological menstrual cycle. Besides, pregnancy rate was slightly higher in the group treated with myo-ins with respect to either placebo or metformin.

Given the ability to induce ovulation in PCOS women, the efficacy of myo-inositol supplementation was also assessed in preparation to ART protocols. A pioneering study by Chiu et al. [28] investigated myo-ins serum levels in women undergoing in vitro fertilization (IVF), one of the most diffused ART. The results evidenced that women whose pregnancy resulted in abortion displayed lower myo-ins levels. Following evidence by the same group [29], it was underlined that patients undergoing IVF who had mature and fertilized oocytes were more likely to have higher myo-ins content in the follicular fluid. Later, Ravanos et al. [30] investigated the relationship between myo-ins/d-chiro-ins ratio in the follicular fluid from egg donors, and the quality of blastocysts after intra-cytoplasmatic sperm injection (ICSI). They found a strong correlation between higher myo-ins content and higher quality embryos, defining myo-ins/d-chiro-ins ratio as a potential marker of oocyte quality. Myo-ins capability to improve fertility was also tested in non-PCOS patients undergoing IVF. The results from these studies are summarized in Table 2. Lisi et al. [31] enrolled women undergoing IVF and divided them into two groups, receiving either 150 IU/die FSH after 400 mcg/die folate for three months, or the same amount of FSH after 400 mcg/die folate and 4000 mg/die myo-ins for three months. Their results underlined that women treated with myo-ins needed a significantly lower FSH amount to reach follicular maturation, requiring on average 2.084 UI as total dose in women treated with myo-ins and 2.479 UI for the control group. A following investigation by Caprio et al. [32] confirmed the results, also finding a significantly higher rate of metaphase II oocytes (80.5%, myo-ins vs. 66.6%, control) and a higher ovarian sensitivity index in women taking myo-ins (1.88 vs. 1.54). These data suggest that myo-ins could be beneficial for all the infertile women undergoing IVF to decrease the total amount of exogenous FSH needed for stimulation and increase the number of retrievable oocytes.

Two following meta-analyses confirmed these results. The meta-analysis by Laganà et al. [33] included studies assessing myo-ins efficiency against a placebo or d-chiro-ins. They confirmed the lower amount of exogenous FSH required to trigger ovulation, with similar duration of ovarian stimulation between women taking myo-ins and controls. Furthermore, as also reported by Colazingari et al. [34], they suggest that myo-ins in PCOS women undergoing IVF promotes the progression of folliculogenesis, contributing to the formation of large follicles, and thus reducing ovarian hyperstimulation risk. The meta-analysis by Zheng et al. [35] investigated the efficiency of myo-ins supplementation on different parameters, including pregnancy and abortion rate, proportion of grade 1 embryos or degenerated oocytes, and exogenous FSH required. Particularly, they highlighted that myo-ins administration increases pregnancy rate and decreases abortion rate with great significance. Moreover, the authors underlined that myo-ins could be helpful in ameliorating grade 1 embryos proportion, while diminishing degenerated oocytes.

### 3.2. Myo-Inositol in the Prevention of Gestational Diabetes Mellitus

**Figure 4:**
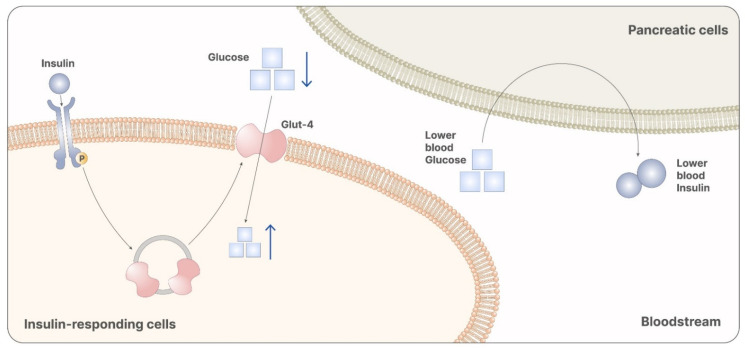
Myo-inositol participates in the signaling cascade of insulin. Particularly, it is involved in the releasing of vesicles containing GLUT-4. GLUT-4 promotes the absorption of glucose from the bloodstream, reducing systemic glycemia. Reduced glycemia is then recognized from pancreatic cells as a stimulus that inhibits insulin release. Therefore, myo-ins administration leads to reduced levels of glucose and insulin, preventing the onset of GDM.

Transmitting the intracellular signal of insulin is one of the most important physiological roles carried out by both myo-ins and d-chiro-ins. Larner et al. [5] identified two inositol glycans that are second messengers of insulin, describing them as containing either d-chiro-ins or myo-ins. The authors also observed that both the inositol glycans act as insulin mimetics when administered in vivo, confirming their involvement in insulin signaling [5]. Based on this evidence, researchers investigated the effects of myo-ins and d-chiro-ins administration in patients with insulin-related pathologies, like diabetes or insulin resistance. Particularly, they found significantly decreased systemic insulin levels in patients treated with either myo-ins or d-chiro-ins, with respect to controls [36]. In particular, the insulin signal mediated by myo-ins allows the release of vesicles containing GLUT-4. On a systemic level, glucose uptake lowers serum glycemia, reducing insulin production (Figure 4) [36]. Comparing the effects of myo-ins and metformin in patients with altered insulin levels, Fruzzetti et al. concluded that myo-ins proved to be as efficient as metformin in lowering insulin levels [37]. Later on, other authors demonstrated that myo-ins is even more efficient than metformin in raising sensitivity to insulin, thus lowering serum insulin levels, homeostatic model assessment of insulin resistance index (HOMA-IR), and LDL cholesterol levels [38]. Furthermore, comparing the risk/benefit ratio of the two molecules, myo-ins clearly represents an alternative to metformin, given also its greater safety and tolerability [38].

On these premises, myo-ins finds interesting applications to prevent pregnancy complications. In fact, insulin status can vary widely during pregnancy, leading to altered maternal–fetal crosstalk and dysregulated fetal growth. Particularly, a greater portion of pregnant women develop insulin resistance, [10] generally resulting in insulin overproduction to decrease glucose levels in the blood. However, such compensatory mechanism fails to occur in some cases. As a consequence, insulin levels remain low and blood glucose levels stay over a safe threshold. This condition is called gestational diabetes mellitus, and it can interfere with the correct progression of the pregnancy [10]. In the mother, high glucose levels can lead to high blood pressure and proteinuria, a clinical picture called pre-eclampsia, which is associated with stillborn babies, miscarriages, and preterm delivery. Pre-eclampsia may also degenerate into eclampsia, a pathology characterized by convulsion seizures with a significant death rate [39]. The occurrence of GDM also results in higher glucose levels in the placenta, exposing the fetus to an unsafe environment. Furthermore, high glucose levels in the maternal blood inhibit placental expression of SMIT and IMPA1, two enzymes responsible for the transport and the synthesis of myo-ins, respectively. This reduces fetal production of myo-ins and its transport from the mother to the fetus, altering inositol signaling in the child too [40]. As suggested by Chu et al. [41], placental myo-ins could exert a protective role against proadipogenic effects of maternal elevated glycemia. Hence, an altered fetal pool of inositols may lead to impaired insulin signaling, resulting in macrosomia, or large-for-gestational-age fetuses. Besides, high glucose levels during pregnancy may result in negative neonatal outcomes, including hypoglycemia. Risk factors associated with GDM onset are overweight condition or obesity, excessive weight gain during pregnancy, or familiar history of diabetes [10]. Interestingly, PCOS is also associated with GDM occurrence. Indeed, GDM is the most frequent pregnancy complication that PCOS women experience, with a three-time higher probability than non-PCOS women, probably due to the pre-existing insulin resistance phenomena [42].

As reported in Table 3, physicians established that myo-ins administration is safe during pregnancy to efficiently prevent GDM. In a retrospective study, D’Anna et al. [43] compared 46 PCOS patients taking 4000 mg myo-ins plus 400 mcg folic acid daily with 37 PCOS patients treated daily with 1.5 mg metformin plus 400 mcg folic acid until evidence of pregnancy. The authors found out that GDM incidence rate was 17.4% in the group treated with myo-ins, and 54% in the control group. This study represents the first insight in myo-ins capability to prevent GDM in PCOS women. Two following randomized and placebo-controlled trials investigated whether myo-ins administration prevents GDM insurgence, studying also related secondary outcomes.

Matarrelli et al. [44] studied myo-ins ability to prevent GDM in non-PCOS women with elevated fasting glucose in the first trimester. Patients were randomized in two groups, taking either 400 mcg/die folic acid or 4000 mg/die myo-ins plus 400 mcg/die folic acid. The authors also evaluated the need for insulin therapy, cases of neonatal hypoglycemia, and gestational age at delivery. At the end of the second trimester, maternal oral glucose tolerance test (OGTT) revealed an abnormal score in 27 out of 38 women in the control group, while in only 2 out of 35 women treated with myo-ins. Eight women in the control group needed insulin intervention, while only one woman in the myo-ins group required treatment with insulin. Ten children belonging to the control group displayed neonatal hypoglycemia, while all children in the study group had normal glycemia. Gestational age at delivery was 37.2 ± 2.04 weeks in the control group, and significantly improved to 39.3 ± 1.6 weeks in the myo-ins group. These findings represent the first evidence that myo-ins may improve glycemic conditions in pregnant women.

In the same year, D’Anna et al. [45] investigated similar parameters in nonobese women with a family history of type 2 diabetes and normal glucose values. A total of 197 women participated in the study, randomized in two groups. The control group was treated with 200 mcg folic acid twice per day, while the study group was treated with 2000 mg myo-ins plus 200 mcg folic acid twice per day. GDM rate in the control group was 15.3%, significantly falling to 6% in the treated group. Furthermore, the authors also highlighted a significant reduction in macrosomia, with seven cases in the control group and none in the treated group, and birthweight, which averaged 3273 ± 504 g in the control group and 3111 ± 447 g in the group treated with myo-ins. These data, as with those from Matarrelli et al., provide high quality evidence on the potential of myo-ins as a preventive treatment in women at risk for GDM.

D’Anna et al. [46] carried out a further study to evaluate whether myo-ins also prevents GDM in obese patients. A total of 220 women with body mass index (BMI) >30 kg/m^2^ were randomized in two groups, treated either with 2000 mg myo-ins plus 200 mcg folic acid twice per day or only with 200 mcg folic acid twice per day. GDM was diagnosed based on OGTT performed between the 24th and the 28th pregnancy week. The authors reported a 14% GDM occurrence in the myo-ins group, compared to 33.6% GDM rate in the control group, confirming the efficiency of myo-ins in the prevention of GDM also in obese patients. The same research group obtained similar results in overweight patients, with a BMI ranging from 25 to 30. In fact, using the same protocol and posology, Santamaria et al. [47] found an 11.6% GDM rate in overweight women treated with myo-ins, with an increase to 27.4% in those treated only with folic acid. Santamaria et al. [48] later analyzed databases from randomized controlled trials involving myo-ins treatment to prevent GDM. This analysis included 595 patients who participated in three different clinical trials, and evaluated secondary outcomes related to GDM. The data show significant reduction in the incidence of preterm delivery (3.4% myo-ins versus 7.6% control), large-for-gestational-age fetuses (4.8% myo-ins versus 8.9% control), and macrosomia (2.1% myo-ins versus 5.3%control). Most importantly, the authors pointed out a significant improvement in GDM incidence rate, falling from 25.3% in the control group to 11% in the treated group. A recent study by Vitale et al. [49] confirmed that myo-ins administration decreases GDM insurgence rate in overweight nonobese pregnant women. Furthermore, they evaluated total body water, along with the extracellular and the intracellular fraction. Notably, they demonstrated that women treated with myo-ins experience a lower increase in body weight and water retention, compared to the placebo group. Indeed, they emphasize that higher total body water and higher body fat relate to higher GDM incidence, suggesting the preventive activity of myo-ins. Taken together, all these studies strongly indicate that myo-ins is also a beneficial treatment in obese and overweight patients, which are known to be at a higher risk for GDM onset. In particular, they evaluate the efficiency of myo-ins to prevent GDM in 631 women, providing high quality data.

Other studies compared the effects of the administration of myo-ins, d-chiro-ins, and the combined therapy on GDM onset rate. Particularly, the study by Celentano et al. [50] included four groups of nonobese singleton pregnant women displaying elevated fasting glucose in the first trimester: (1) controls, treated with 400 mcg/die folic acid; (2) patients treated with 4000 mg/die myo-ins plus 400 mcg/die folic acid; (3) patients taking 500 mg/die d-chiro-ins plus 400 mcg/die folic acid; (4) patients treated with the 40:1 combination of myo-ins and d-chiro-ins (1100 mg/die myo-ins plus 27.6 mg/die d-chiro-ins). The authors reported a significant difference in abnormal maternal OGTT, occurring in 61.5% of the controls, 5.1% of women treated with myo-ins, 34.4% of women treated with d-chiro-ins, and 38.2% of women who received the combined treatment. Furthermore, they observed a significant reduction in episodes of neonatal hypoglycemia following myo-ins treatment. In fact, all children born from mothers taking myo-ins were healthy, while hypoglycemia occurred in 21.1% of the control group, 15.6% of the d-chiro-ins group, and 8.8% of the group treated with inositol combination. Later, Vitagliano et al. [51] carried out a meta-analysis comparing the efficiency of myo-ins with the combined myo-ins and d-chiro-ins treatment (40:1 ratio). The authors analyzed fasting OGTT, 1 h OGTT, and 2 h OGTT, demonstrating a better efficiency of myo-ins treatment in keeping all the OGTT parameters within the safety threshold. The authors finally highlight that both the treatments are likely to improve hypertensive disorders and preterm delivery incidence.

### 3.3. Myo-Inositol in the Prevention of Neural Tube Defects

Neural tube formation is a critical phase during embryogenesis. The process is very complex, and part of the mechanisms involved are still unclear to this day. The fine genetic regulation is of primary importance, being that the formation is strongly dependent on correct signals from other tissues [11]. Currently, up to 0.5% of pregnancies results in fetuses carrying NTDs [52]. Generally, the most delicate moment during neural tube formation is the closure, which may result in NTDs, like spina bifida or anencephaly, if not properly regulated [18]. Spina bifida comes into several manifestations, most of which are compatible with life. Myelomeningocele is the most severe of them, and also one of the most common congenital malformations. Its etiology strongly depends on genetic alterations, even if up to 40% of cases have environmental causes [53]. According to National Institutes of Health (NIH), NTDs are more likely to occur in women with previous pregnancies affected by NTDs, with 4% chance after the first NTD episode and up to 10% after the second. People with a history of spina bifida face the same risk when deciding to conceive [54]. Other risk factors include maternal obesity, maternal uncontrolled diabetes, and maternal consumption of drugs with teratogenic activity, such as anticonvulsants, like valproate or lithium carbonate [55,56].

**Figure 5:**
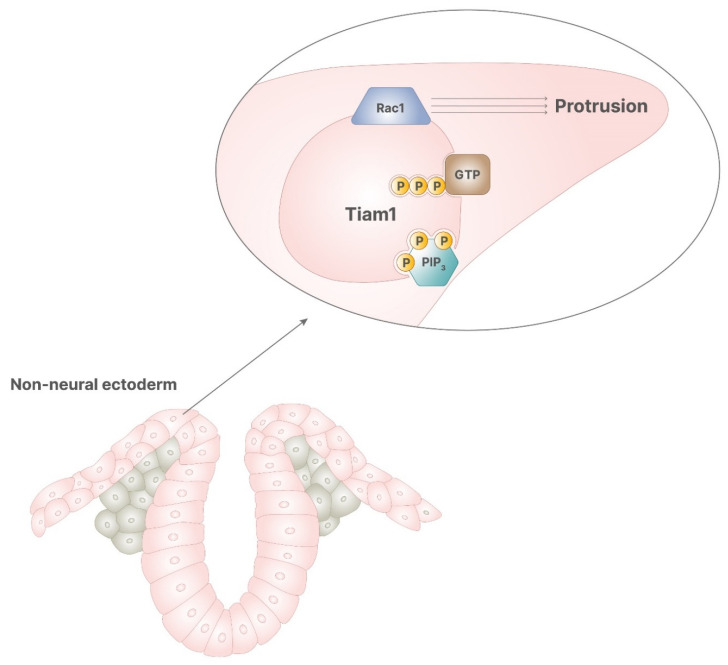
In the correct signaling of retinoic acid, PIP3 stimulates the activity of Tiam1, which has GDP/GTP exchange activity, enabling the function of Rac1. This process allows the cytoskeletal rearrangement, required for cellular protrusion, which leads to the correct closure of neural tube.

In the prevention of NTDs, folate supplementation is the first-choice approach, due to the beneficial effects observed during the years. Besides, folate induces the expression of Rac1 and RhoA, two G proteins involved in cytoskeleton rearrangement, and cellular motility and invasiveness [57]. However, some NTDs (about 30%) are not responsive to folate. This phenomenon, known as folate resistance, was detected in humans and studied in the curly tail murine model, displaying NTD predisposition and folate resistance [58]. As inositol deficiency exacerbates the incidence of NTDs in such a murine model [59], researchers evaluated the effects of myo-ins supplementation as an alternative to folate. They found that such a treatment reduced NTD occurrence in pregnant mice, showing a prevention rate of up to 70%. Moreover, the authors also observed that protein kinase C (PKC) stimulation mimicked the effects of inositol administration on NTD rate [58]. In fact, myo-ins mediates the activity of PKC-γ and PKC-ζ, both necessary in the prevention of NTDs [8]. Inositol administration also induces the expression of retinoic acid receptor in the caudal portion of the embryos [58]. Retinoic acid signaling, indeed, induces massive cytoskeletal rearrangement through Rac1 activity, required for the correct neural tube closure (Figure 5) [60]. Therefore, by increasing the intracellular pool of inositol phosphates, myo-ins administration enhances PKC activity and restores the correct retinoic acid signaling, preventing NTDs. From a clinical perspective, low blood levels of maternal myo-ins are related to a higher risk of NTDs, confirming preclinical evidence [61]. As D’Souza et al. [62] suggested, the yolk sac plays an important role in the mother-to-fetus inositol delivery. In fact, in the first trimester, maternal blood does not directly come in contact with the placenta, but flows through intercellular spaces, accessing the intervillous space surrounding the placenta and reaching the yolk sac. In turn, the yolk sac is connected with embryonic gut and vitelline circulation, allowing the passage of nutrients from maternal serum to the embryo.

On these premises, physicians investigated the preventive effects of myo-ins also in humans. These studies are summarized in Table 4. Cavalli et al. [63] provided the first clinical evidence with a study involving three women with two previous NTD episodes. Interestingly, these patients were treated with folate for at least one of their two NTD episodes and were, thus, considered folate resistant. The authors recommended the intake of 500 mg inositol plus 5 mg folic acid daily, starting from three months before conception and until the end of the second month of pregnancy. None of the fetuses were diagnosed with NTDs, resulting lately in three healthy babies. Two of the three women decided to conceive again, undergoing inositol plus folic acid treatment and giving birth to two healthy babies. Remarkably, the authors report no adverse effect. However, because of the small sample size and the absence of controls, the same group expanded these results years later during a following study [64]. An additional nine women, with previous NTD episodes despite correct folate intake, were enrolled in the study. They received 1000 mg inositol plus 5 mg folic acid daily, starting at least two months before conception until at least the end of the second month of pregnancy. Among 11 newborns, none suffered from NTDs, confirming the hypothesis that myo-ins could provide an effective adjunction to folate treatment or a valid alternative in folate resistant patients. Years later, Greene et al. [65] carried out a breakthrough study on the efficiency of myo-ins in NTD prevention. In this randomized, placebo-controlled trial, they recruited 48 patients with a previous history of NTDs and divided them into two groups: intervention (23 women) and control (25 women). Patients in the intervention group received 500 mg inositol two times a day, while the control group received placebo. All the women were treated with 5 mg folic acid daily. Fourteen pregnancies occurred in the intervention group, reporting no NTD episodes; while, among 19 pregnancies in the placebo group, one was terminated due to NTD. The study also included an additional 22 women who refused randomization, 19 of which chose to take myo-ins and 3 opted for only folic acid supplementation. In the first group, all women conceived and no episodes of NTDs occurred in all the physiological pregnancies, resulting in healthy children. Moreover, in the second group, all the women conceived, but NTDs occurred in two pregnancies, thus resulting in termination. Even if the sample size is small, the data indicate that women who do not take myo-ins have a 13% NTD recurrence, comparable with NIH estimates. Interestingly, myo-ins supplementation seems to reduce the incidence of NTDs to a much greater extent.

The clinical and preclinical trials demonstrate that the proper intake of myo-ins is of primary importance in the correct development of the fetus. Particularly, these studies highlight that myo-ins supplementation may prevent NTDs occurrence in case of inositol deficiency or folate resistance.

## 4. Conclusions

Myo-ins plays a crucial role in fertility and in supporting physiological gestation. As second messenger of gonadotropins and insulin, it guarantees the correct development of oocytes and the progression of embryogenesis. Available evidence demonstrates that the supplementation with myo-ins enhances the probability of achieving pregnancy and reduces the onset of adverse maternal effects and neonatal outcomes. Specifically, in the pursuit of pregnancy, myo-ins proved to induce ovulation and restore the physiological menstrual cycle in infertile women, particularly in those with PCOS. Moreover, myo-ins supplementation is also useful for women undergoing ART, improving oocyte and embryo quality. During pregnancy, myo-ins reduces the risk of altered metabolic conditions like GDM, which may lead to miscarriages or stillborn babies. In addition, myo-ins seems to reduce the risk of NTDs in folate-resistant women.

## Figures and Tables

**Table 1:** Summary of the studies involving myo-inositol as a treatment for ovulation induction, regularization of menstrual cycle or in the pursuit of pregnancy.

Study	Patients	Protocol	Findings
Gerli et al. 2003 [22]	283 PCOS women with oligomenorrhea or amenorrhea	Randomized, placebo-controlled, treatment with 100 mg twice a day for 14 weeks	Ovulation rate: 23% in the treatment group versus 13% in the control group
Costantino et al. 2009 [23]	42 PCOS women with oligomenorrhea, high serum free testosterone, and/or hirsutism	Double-blind, randomized, placebo-controlled, treatment with 2000 mg twice a day for 6 weeks	Ovulation rate: 69.5% in the treatment group versus 21% in the control group Progesterone peak value: 15.1 ng/mL in the treatment group versus 6.6 ng/mL in the control gorup
Papaleo et al. 2007 [24]	25 infertile women showing PCOS as the only apparent cause of infertility	Open-label treatment with 2000 mg twice a day for 6 months	Serum progesterone: 1.8 ± 0.7 ng/mL at baseline versus 10.5 ± 1.8 ng/mL after treatment Percentage of patients with at least one menstrual cycle: 0% at baseline versus 88% after treatment Percentage of patients with regular ovulations: 0% at baseline versus 72% after treatment Percentage of pregnancy achieved during the treatment: 40%
Raffone et al. 2010 [25]	120 anovulatory, infertile PCOS women	Randomized treatment for 6 months with 4000 mg/die myo-inositol versus 1500 mg/die metformin; nonpregnant patients from both groups underwent 37.5 U/die FSH treatment for a maximum of three times	Pregnancy rate after the first treatment: 26.1% in the metformin group versus 28.9% in the myo-inositol group Total pregnancy rate following FSH treatment: 36.6% in the metformin group versus 48.4% in the myo-inositol group
Allah et al. 2020 [26]	140 sub-fertile PCOS women	Open-label treatment with 2000 mg per day for 6 months	Percentage of patients with regular menstrual cycle: 0% at baseline versus 24.3% after three months versus 53.6% after six months Percentage of ovulating patients: 0% at baseline versus 38.6% after three months versus 72.1% after six months

**Table 2:** Summary of the studies involving myo-inositol as a pretreatment for women undergoing IVF.

Study	Patients	Protocol	Findings
Lisi et al. 2012 [31]	100 non-PCOS women with basal FSH <10 mUI/mL	Randomized, controlled treatment with 2000 mg twice a day for 3 months	Exogenous FSH required to reach follicular maturation: 2.084 UI in the treatment group versus 2.479 UI in the control group
Caprio et al. 2015 [32]	76 non-PCOS infertile women	Controlled treatment with 4000 mg/day for 3 months	Percentage of metaphase II oocytes: 80.5% in the treatment group versus 66.6% in the control group Ovarian sensitivity index: 1.88 ± 0.81 in the treatment group versus 1.54 ± 0.65 in the control group

**Table 3:** Studies involving myo-inositol as a preventive treatment against GDM onset.

Study	Patients	Protocol	Findings
D’Anna et al. 2012 [43]	98 pregnant PCOS women	Retrospective study of women taking 4000 mg/die myo-inositol throughout the whole pregnancy versus 1500 mg/die metformin until pregnancy occurs	GDM incidence: 17.4% in the treatment group versus 54% in the control group
Matarrelli et al. 2013 [44]	73 pregnant women, or intended to become pregnant, with glycemia ≥5.1 mmol/L or 92 mg/dL and ≤7.0 mmol/L or 126 mg/dL	Randomized, double-blind, placebo-controlled treatment with 4000 mg/die for the entire pregnancy	GDM incidence: 6% in the treatment group versus 71% in the control group Need for insulin: 3% in the treatment group versus 21% in the control group Neonatal hypoglycemia: 0% in the treatment group versus 26% in the control group
D’Anna et al. 2013 [45]	197 pregnant women with a parent with type 2 diabetes	Randomized, placebo-controlled treatment with 2000 mg twice per day	GDM incidence: 6% in the treatment group versus 15.3% in the control group Macrosomia cases: 0 in the treatment group versus 7 in the control group Birthweight: 3111 ± 447 g in the treatment group versus 3273 ± 504 g in the control group
D’Anna et al. 2015 [46]	201 pregnant women with BMI ≥ 30 kg/m^2^	Randomized, placebo-controlled treatment with 2000 mg twice per day	GDM incidence: 14% in the treatment group versus 33.6% in the control group
Santamaria et al. 2016 [47]	207 women with BMI > 25 and <30 kg/m^2^ and fasting plasma glucose ≤126 mg/dL and/or glycemia <200 mg/dL	Randomized, placebo-controlled treatment with 2000 mg twice per day from the first trimester to the end of the pregnancy	GDM incidence: 11.6% in the treatment group versus 27.4% in the control group
Vitale et al. 2020 [49]	223 women with BMI > 25 and <30 kg/m^2^ and fasting plasma glucose ≤126 mg/dL and/or glycemia <200 mg/dL	Randomized, placebo-controlled treatment with 2000 mg twice per day from the first trimester to three weeks after delivery	GDM incidence: 8.2% in the treatment group versus 21.2% in the control group Weight gain: 8.33 ± 2.47 kg in the treatment group versus 9.31 ± 2.66 kg in the control group Total body water in the third trimester: 51.30 ± 4.65 L in the treatment group versus 53.82 ± 4.13 L in the control group
Celentano et al. 2018 [50]	157 nonobese pregnant women with fasting glycemia ≥5.1 mmol/L or 92 mg/dL and <7.0 mmol/L or 126 mg/dL	Randomized, placebo-controlled treatment for the entire pregnancy with 2000 mg myo-inositol twice per day, 500 mg d-chiro-inositol per day, or 13.8 mg d-chiro-inositol and 550 mg myo-inositol twice per day	GDM incidence: 5.1% in the myo-inositol group versus 34.4% in the d-chiro-inositol group versus 38.2% in the combined group versus 61.5% in the control group Neonatal hypoglycemia: 0% in the myo-inositol group versus 15.6% in the d-chiro-inositol group versus 8.8% in the combined treatment group versus 21.1% in the control group

**Table 4:** Studies involving myo-inositol as a preventive treatment against NTDs in women with a previous pregnancy affected by NTD despite folate supplementation.

Study	Patients	Protocol	Findings
Cavalli et al. 2008 [63]	3 women with at least one previous pregnancy affected by folate-resistant NTD	Open-label treatment with 500 mg per day from at least two months before and until 60 days after conception	NTD incidence: 0%
Cavalli et al. 2011 [64]	9 women with at least one previous pregnancy affected by folate-resistant NTD	Open-label treatment with 1000 mg per day from at least two months before and until 60 days after conception	NTD incidence: 0%
Greene et al. 2016 [65]	47 randomized and 22 non-randomized women with at least one previous pregnancy affected by NTD	Randomized, double-blind, placebo-controlled treatment with 500 mg twice per day; women who declined randomization decided to take myo-inositol plus folic acid (19 patients), or folic acid only (3 patients)	NTD incidence in randomized patients: 0% in the treatment group versus 5.3% in the control group NTD cases in the non-randomized patients: 0 in the myo-inositol plus folic acid group versus 2 in the folic acid alone group Overall NTD incidence: 0% in the treatment group versus 13.63% in the control group

## Data Availability

Data sharing is not applicable to this article.
